# Gene Expression of ANP, BNP and ET-1 in the Heart of Rats during Pulmonary Embolism

**DOI:** 10.1371/journal.pone.0011111

**Published:** 2010-06-14

**Authors:** Henrik Gutte, Jytte Oxbøl, Ulrik Sloth Kristoffersen, Jann Mortensen, Andreas Kjær

**Affiliations:** 1 Department of Clinical Physiology, Nuclear Medicine and PET, Rigshospitalet, Copenhagen University Hospital, Copenhagen, Denmark; 2 Cluster for Molecular Imaging, Faculty of Health Sciences, University of Copenhagen, Copenhagen, Denmark; University of Cincinnati, United States of America

## Abstract

**Aims:**

Atrial natriuretic petide (ANP), brain natriuretic peptide (BNP) and endothelin-1 (ET-1) may reflect the severity of right ventricular dysfunction (RVD) in patients with pulmonary embolism (PE). The exact nature and source of BNP, ANP and ET-1 expression and secretion following PE has not previously been studied.

**Methods and Results:**

Polystyrene microparticles were injected to induce PE in rats. Gene expression of BNP, ANP and ET-1 were determined in the 4 cardiac chambers by quantitative real time polymerase chain reaction (QPCR). Plasma levels of ANP, BNP, ET-1 and cardiac troponin I (TNI) were measured in plasma. PE dose-dependently increased gene expression of ANP and BNP in the right ventricle (RV) and increased gene expression of ANP in the right atrium (RA). In contrast PE dose-dependently decreased BNP gene expression in both the left ventricle (LV) and the left atrium (LA). Plasma levels of BNP, TNI and ET-1 levels dose-dependently increased with the degree of PE.

**Conclusion:**

We found a close correlation between PE degree and gene-expression of ANP, and BNP in the cardiac chambers with a selective increase in the right chambers of the heart.

The present data supports the idea of natriuretic peptides as valuable biomarkers of RVD in PE.

## Introduction

Cardiovascular homeostasis and function results from complex interactions of various regulatory factors. Among these are the cardiac natriuretic peptides (atrial natriuretic petide (ANP) and brain natriuretic peptide (BNP) as well as endothelin-1 (ET-1). The cardiac gene expression and plasma level of ANP and BNP and ET-1 has been subject of intense investigation concerning their potential role and secretion in response to acute pulmonary embolism (PE). The natriuretic peptides are biologically active substances capable of inducing diuresis, natriuresis and reducing systemic blood pressure. Plasma concentration of the hormones are augmented in patients with chronic heart failure and predicts left ventricular (LV) ejection faction (EF), mortality and morbidity in these patients[1]. BNP level is also increased in valvular diseases, chronic pulmonary hypertension and chronic right ventricular dysfunction (RVD)[2]. BNP in particular originates from the ventricles of the heart and is released in response to increased filling pressure[3]. ANP is released by distension, primarily from atrial tissue in healthy subjects. ET-1 is released from endothelial cells in the pulmonary circulation. ET-1 plasma levels correlate with the pulmonary pressure, and abnormalities in the circulating levels of ET-1 have been reported in an experimental PE model[4].

Also in patients ANP, BNP and ET-1 are released in response to volume expansion and pressure overload and may identify RVD[5] and have been shown to correlate with the severity of RVD in PE patients[6]. Patients with RVD after PE have a worse prognosis and may have increased mortality compared with those who have a normal RV function[7] and these biomarkers may therefore help to stratify these patients[8].

Previous studies have shown that the gene expression of BNP, ANP and ET-1 is induced both in the RV and LV in a chronic model of RVD[9], [10]. But no one has so far showed the origin and extent of gene expression of these hormones in response to acute PE.

Our hypothesis was therefore that the gene expression of ANP, ET-1 and BNP was upregulated in the RV during PE and the hormone plasma concentration and gene expression was correlated to the extent of PE.

Therefore, the aim of our study was to quantitatively establish the gene expression profiles of BNP, ANP and ET-1 in all 4 chambers of the heart in rats during various degrees of PE. To do so, we used quantitative real-time polymerase chain reaction (QPCR) to measure the levels of the gene expression during various degrees of experimental PE in a rat model of PE. In addition, the associations between the gene expression profiles of the hormones were compared with plasma levels of the hormones to evaluate the association between the soluble biomarker and local gene expression levels.

## Materials and Methods

Animal care and all experimental procedures were performed under the approval of the Danish Animal Welfare Council (2006/561-1124). Experiments were performed using male Sprague-Dawley rats (Charles River, Sulzfeld, Germany) weighing between 328–400 g. The rats were housed 2 animals per cage, with a 12∶12-h light-dark cycle and received standard rat chow and water *ad libitum* throughout the course of experiments.

### Pulmonary embolism model

Polystyrene microspheres were used to create pulmonary embolism as previously described[11]. Microspheres (25±1 µm; Duke Scientific catalogue no. 7525, Fremont, Ca, USA) were sterilized with 70% ethanol, washed with sterile 0.01% Tween 20, and resuspended in 0.01% sterile Tween 20 to produce a 10% suspension (10.39 million beads/mL). All animals were anesthetized with 3% sevoflurane (Abbott Scandinavia AB, Solna, Sweden) mixed with 35% O_2_ in N_2_ by breathing. The rats were randomly assigned to 4 groups. Microspheres were injected via a tail vein to produce mild (0.87 million beads/100 g body weight, referred to as PE 0.87), moderate (1.30 million beads/100 g body weight, referred to as PE 1.30) and severe PE (1.95 million beads/100 g body weight, referred to as PE 1.95). Sham animals received vehicle (0.01% Tween 20, 0.15 mL/100 g body weight). After 16 hours of treatment, all animals were anesthetized with 3% sevoflurane (Abbott Scandinavia AB, Solna, Sweden) mixed with 35% O_2_ in N_2_ by breathing and subsequently decapitated. Blood was collected in EDTA containers with 500 µL Trasylol, 10.000 KIE/mL (Bayer HealthCare, Leverkusen, Germany), centrifuged at 2,000×g for 15 min, and plasma was transferred to a fresh tube and stored at −80°C until analyzed.

### Tissue dissection, RNA extraction and Reverse Transcription

The heart tissue was removed, dissected into RV, LV, right atrium (RA), left atrium (LA) and interventricular septum (IVS) and weighed and hereafter placed in RNA*later*® (Ambion (Europe) Limited, Cambridgeshire, UK), and stored at 4°C. The following day the RNA*later*® was removed and the samples were stored at −80°C for later use.

Total RNA (20–30 mg) was isolated using NucleoSpin RNA II Kit (Macherey-Nagel, Düren, Germany). The total RNA concentration was measured on a Bioanalyzer 2100 (Agilent Technologies, Waldbronn, Germany); using RNA 6000 Nano kit (Agilent, Technologies, Waldbronn, Germany). Total RNA was reverse transcribed using AffinityScript QPCR cDNA Synthesis Kit (Stratagene, La Jolla, CA, USA). The cDNA was immediately cooled down and stored at −20°C.

### Quantitative real-time PCR

The primers and LNA (Locked Nucleic Acid) fluorescent probes for the three genes of interest BNP (NM_031545), ET-1 (NM_012548), ANP (NM_012612) and the housekeeping gene TBP ((TATAA-box Binding Protein, NM_001004198) were designed using the software Beacon Designer (version 6.0, Premier BioSoft, Palo Alto, CA, USA). The primer and probe sequences were purchased from Sigma-Genesis (Sigma-Aldrich, St-Louis, MO, USA). The primers and probes are shown in Table 1. Each PCR reaction was optimized regarding forward primer (FP), reverse primer (RP) and probe concentrations (final): BNP FP/RP 300/600 nM, probe 300 nM; ANP FP/RP 300/300 nM, probe 200 nM; ET-1 FP/RP 300/600 nM, probe 300 nM and TBP FP/RP 300/300 nM, probe 100 nM.

**Table 1:** Primer and probe sequences.

Gene	Forward primer, 5′ - 3′	Reverse primer, 5′- 3′	5′-Flourophore	LNA probe, 5′- 3′	3′- Quencher	Amplicon length
ANP	gaggagaagatgccggtag	ctagagagggagctaagtg	FAM	cgcTtcAtcGgtCtgct	BHQ-1	96 bp
BNP	tgattctgctcctgcttttc	gtggattgttctggagactg	FAM	taaTctGtcGccGctgg	BHQ-1	91 bp
ET-1	tgattctcttgcctcttcttg	tatggaatctcctggctctc	FAM	ccaCtcAggAatGgcac	BHQ-1	110 bp
TBP	tgcgttgatcttcagttctg	cttgctgctagtctggattg	HEX	ctcTtgGctCctGtgc	BHQ-1	75 bp

The gene quantification was performed on an Mx3000P® QPCR instrument (Stratagene, La Jolla, CA, USA). The three genes of interest were quantified in duplex assays with TBP. All samples were run in triplicates in a total volume of 25 µl using 1 µl cDNA. Brilliant QPCR Core Reagent Kit (Stratagene) was used. Optimization of the assays resulted in a 50% increase of both dNTP and Taq DNA Polymerase and an MgCl_2_ concentration of 5.5 mM. The thermal profile used was: 1 cycle at 95°C for 10 min (denaturizing), 45 cycles at 95°C for 30 sec and 60°C for 1 min (annealing/elongation).

The optimal housekeeping gene for this study was selected from a panel of 8 common rat endogenous genes (primer-sets from PrimerDesign, Southampton, UK). All genes were tested in tissues from rat hearts representing the 4 groups: 1 treated and 3 untreated groups (n = 10 in each group). By using NormFinder algorithm[12] the overall most stable housekeeping gene was found to be TBP.

Relative quantification of the genes was calculated using the comparative method (2^−ΔΔCt^)[13]. Beside TBP as a housekeeping gene, we also included a calibrator group represented by untreated animals. The efficiency of the PCR (E) was calculated from 5-fold dilution curves for each gene in the PCR reaction. By replacing 2 with (E+1) in the expression 2^−ΔΔCt^, we thereby compensated for variations in the PCR reactions.

To estimate the relative expression of gene of interest in the different cardiac chambers RV was used as calibrator and compared to LV, RA and LA.

### Blood sample analysis

TNI and BNP was measured by an automated two-site sandwich immunoassay technique using chemiluminescence (Siemens, ADVIA Centaur, Leverkusen, Germany).

ANP was measured using an ELISA kit and the lower detection limit for NT-pro-ANP (1–98) was 0.05 nmol/L and the intra- and interobserver CV were 2% and 4%, respectively.

The lower detection limit for NT-pro-ANP (1–98) was 0.05 nmol/L and the intra- and interassay coefficients of variation were 2% and 4%, respectively.

The sensitivity of the Cardiac Troponon I (TNI) assay was 0.015 ng/ml and an interassay coefficient of variation was 10%.

BNP was analyzed using a kit for rats (BNP-45 EIA kit, Phoenix Pharmaceuticals Ca, USA). The sensitivity of the assay was 0.3 ng/ml and the intra- and interassay coefficients of variation were <5% and <14%, respectively.

ET-1 was measured with enzyme-linked immunosorbent assay (ELISA) kits (Biomedica, Eching, Germany). The assay measures the physiologically active ET peptide (1–21). Lower detection limit was 0.02 fmol/ml and the intra- and interassay coefficients of variation were 4% and 6%, respectively.

### Statistical analysis

A one-sample Kolmogorov-Smirnov procedure was performed on all variables to test the assumption of normal distribution. The hormone values were not found to be normal distributed and accordingly log_10_ transformation was performed leading to normal distribution. Gene expression data are presented as mean ± SEM relative to the control for each of the 4 chambers to show the effect of PE. For comparison of relative gene expression in the 4 chambers, RV was used as reference. Comparisons between expressions in chambers were made by paired t-test followed by Bonferroni correction for multiple comparisons. P<0.05 was considered significant. All statistical analyses were performed using SPSS 15.0 (Chicago, IL, USA).

## Results

Weights of the cardiac chambers and rats are presented in table 2. There were no significant differences between the groups of rats.

**Table 2:** Rat weight and weight of the heart chambers.

	BW (g)	HW (g)	LV (g)	RV (g)	IVS (g)	HW/BW (%)	RV/(LV+IVS)	RV/BW (%)
Control (n = 10)	354±13	0.94±0.06	0.49±0.05	0.22±0.03	0.23±0.03	0.26±0.01	0.30±0.04	0.061±0.01
PE 0.87 (n = 10)	368±15	0.93±0.06	0.51±0.04	0.21±0.03	0.21±0.03	0.25±0.02	0.29±0.04	0.056±0.01
PE 1.30 (n = 10)	357±20	0.87±0.10	0.47±0.06	0.21±0.05	0.19±0.04	0.24±0.03	0.33±0.11	0.059±0.02
PE 1.90 (n = 10)	363±11	0.87±0.10	0.45±0.07	0.22±0.04	0.20±0.03	0.24±0.03	0.33±0.07	0.060±0.01

There were no significant differences between the rats. BW: body weight, HW: heart weight, LV: left ventricle, RV: right ventricle, RA: right atrium, LA: left atrium. IVS: interventriclular septum. Mean± SD.

### PE induced gene expression

#### BNP (figure 1)

PE dose-dependently increased BNP gene expression in the RV (2–6 fold; p<0.05-0.001). In contrast PE dose-dependently decreased BNP gene expression in both the LV (0.6-0.5 fold, p<0.001) and the LA (0.8-0.3, p<0.01-0.001). No major changes were seen in the RA.

**Figure 1:**
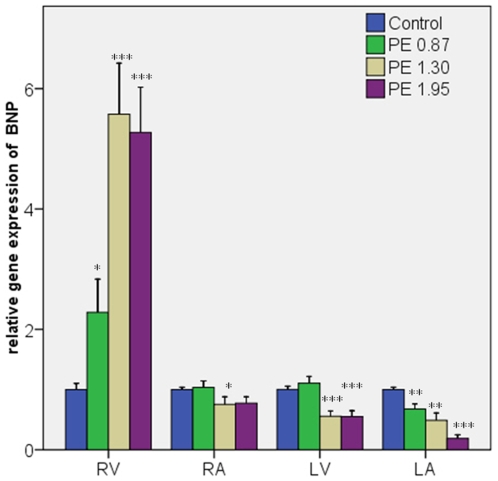
BNP gene expression in the cardiac chambers. Sample sizes were 10 rats in each group. Error bars indicate SEM. RV: right ventricle, RA: right atrium, LV: left ventricle, LA: left atrium. TBP: TATA-box binding protein. *P<0.05; **p<0.01; ***p<0.001 compared to the control group.

#### ANP (Figure 2)

PE dose-dependently increased gene-expression of ANP in the RV 8–9 fold (p<0.05) and in the RA 1.5 fold (p<0.001) compared to the control group. There was an increase of ANP gene expression in LV in the PE 1.30 group (p<0.05). No major changes were seen in the LA.

**Figure 2:**
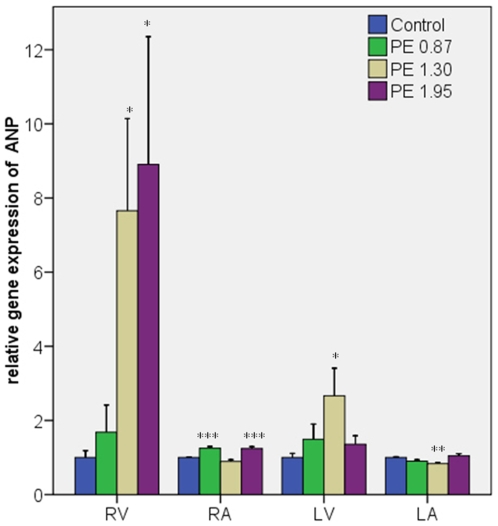
ANP gene expression in the cardiac chambers. Sample sizes were 10 rats in each group. Error bars indicate SEM. RV: right ventricle, RA: right atrium, LV: left ventricle, LA: left atrium. TBP: TATA-box binding protein. *P<0.05; **p<0.01; ***p<0.001 compared to the control group.

#### ET-1

No systematic changes were seen in ET-1 gene expression in the RA, LA, LV and RV during PE (data not shown).

### Relative gene-expression levels in the cardiac chambers

#### BNP

In the control group BNP gene expression was 18 fold higher in the RA and 6–7 fold higher in LV and LA compared to RV expression (p<0.001).

In the PE 0.87 group BNP gene expression in RA was 9 fold higher compared to the RV, LV and LA group (p<0.001).

In the PE 1.30 group BNP gene expression was 3.5 fold higher (p<0.01) in the RA compared to LA and RV.

In PE 1.95 group the RA BNP gene expression was only 1.5 fold higher compared to RV.

#### ANP

ANP was predominately expressed in the LA and the RA in both the control and the PE treated groups compared to the RV (300–1200 fold; p<0.001). The ANP gene-expression in the atria compared to the ventricles was decreased as the degree of PE increased.

#### ET-1

ET-1 mRNA were primarily expressed in the LA (4 fold), LV (2 fold) and RA (4.5 fold) in the control group compared to the gene expression in the RV (p<0.001). This did not change during PE p<0.001-0.01).

### Plasma levels of pro-ANP, TN, ET-1 and BNP

**Figure 3:**
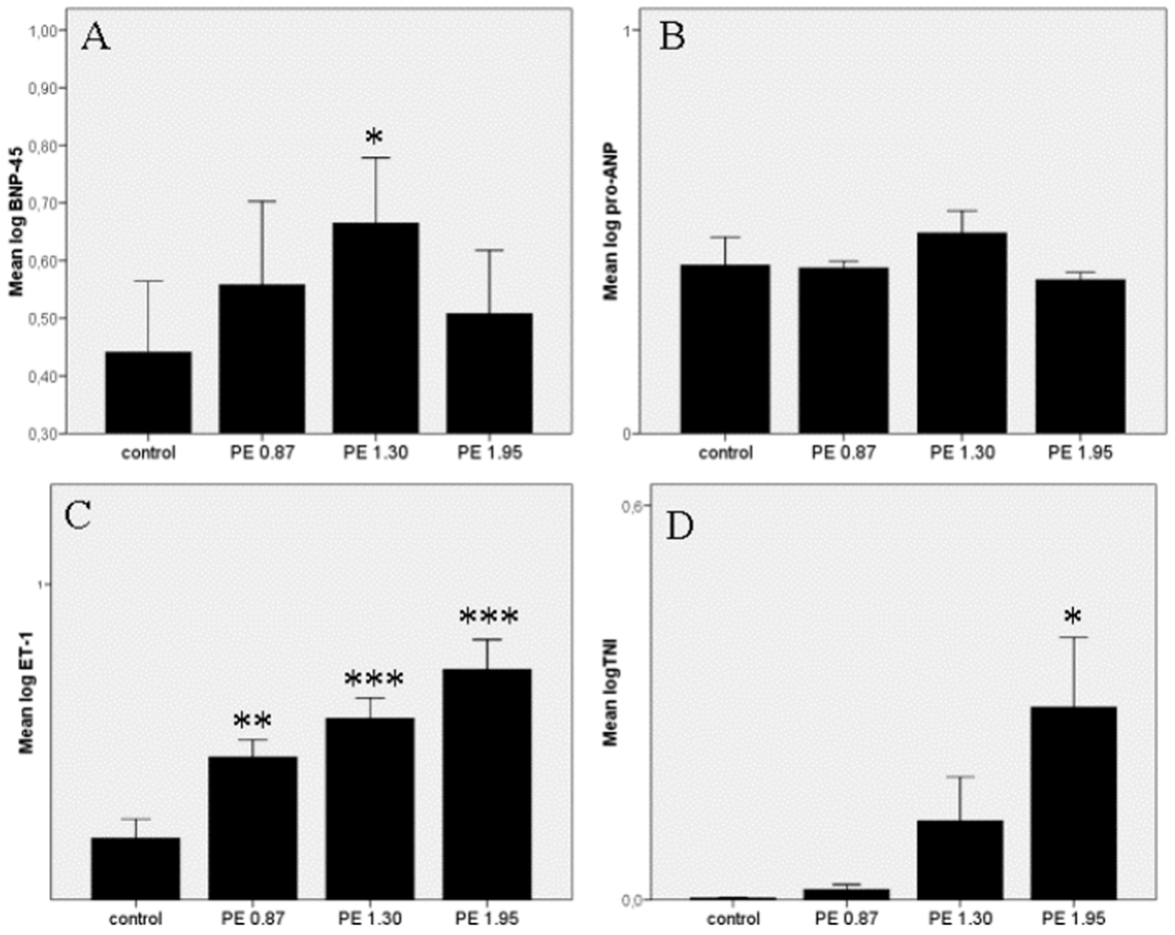
Plasma hormone levels of BNP, pro-ANP, ET-1 and TNI.

Plasma levels pro-ANP, TNI, ET-1 and BNP are shown in Figure 3.

Plasma BNP level (panel A) showed a significant increase from control group to PE 1.30 and a decrease from PE 1.30 to 1.95.

There were no significant changes in the ANP plasma levels (panel B).

Plasma ET-1 level (panel C) and TNI (panel D) significantly and dose-dependently increased in the groups with PE.

## Discussion

In the present study we measured gene expression of ANP, BNP and ET-1 in the cardiac atria and ventricles in response to graded acute PE in an animal model. To our knowledge, this has not previously been done.

We found that gene expression of ANP and BNP were strongly and dose dependently up-regulated in the RV during PE. Both ANP and BNP gene expression reached maximum at PE 1.3 with no further increase at PE 1.95. Therefore, it seems that gene expression in RV was maximally stimulated in our model already at PE 1.3. In addition, we found that the gene expression of BNP was down-regulated in both left chambers in animals with moderate and severe PE.

Comparing BNP gene expression in the control groups the highest level of BNP expression was found in the RA followed by LV. This was also shown in previous studies where the order of the BNP mRNA concentration in the rat heart was RA>LA>LV>RV[14] and with levels in the LV being approximately three-fold lower than the atria in normal functioning hearts[15]. During PE the BNP gene expression increases in the RV and decreases in the LV thereby making the contribution from the RV relatively more important. In chronic left heart failure there is an up-regulation of ventricular BNP secretion[16]and the LV becomes the dominant chamber for peptide secretion[17]. Taking the weight of the ventricles into account, 70% of all cardiac plasma BNP derives from the ventricles under normal conditions and up to 88% under pathophysiological conditions[18], [19]. Plasma hormone levels of BNP were dose-dependently increased in the RV in the PE model. This also supports the idea that RV is the dominant chamber in the secretion of BNP in PE. The model showed that the cardiac gene responses seem to be maximal with the PE 1.3 dose of microspheres, without further increase in the most severe group of PE 1.95. In contrast with circulating BNP, the ET-1 levels continued to increase in the PE 1.95 group beyond those observed in the PE 1.30 group. So, this decrease in the circulating BNP levels was not due to limited progression in severity of the model. We speculate whether BNP was released and peaked at an earlier time point in the severe group and with depleted stores and gene expression already at maximum this would lead to the observed circulating BNP level.

In the control group and during PE we found that the ANP gene expression was expressed higher in the atria than in the ventricles. Based on gene expression and chamber weights, our data indicate that atria are the major source of ANP in the control rats and during PE.

Our results show that ANP is mainly expressed in the atria and is up-regulated on the right side of the heart in rats with PE. It has previous been shown that ANP mRNA in the RV was increased in an animal model of chronic pulmonary hypertension with RV hypertrophy[20]. The present study is to the best of our knowledge the first that uses QPCR on atrial tissue in an animal model of acute PE. Previous studies on tissue specific expression of the ANP gene have shown high-level expression in the RA and virtually undetectable amounts of ANP mRNA and peptides in the LV of healthy adult mammals[18] which was also confirmed in our study. Over 90% of ANP released from the normal adult heart originates from the atria[21]. ANP hormone level was unaffected during PE. ANP is stored in atrial secretory granules and stretch enhances the secretion. The depletable nature of this pool together with low weight of the atria might explain the lack of release of ANP during PE[22].

PE did not systematically affect gene expression of ET-1. ET-1 in the heart was expressed higher in the atria than in the ventricles. Plasma hormone levels were dose-dependently increased in ET-1 in the PE model. There was no association between the level of ET-1 in plasma and gene expression in the cardiac chambers, probably because ET-1 is mainly synthesized and secreted from the pulmonary endothelial cells rather than from the heart[23].

TNI plasma level was dose-dependently increased in the PE model. TNI is released upon myocardial damage and it has been speculated whether TNI plasma level could be used as a prognostic index in patients with PE[24].

Injection of polystyrene microparticles has been a model for experimental PE producing vascular occlusion with physiological alterations similar to PE as observed in humans and causing RVD. The method allows accurate grading of the vascular occlusion. It has previously been demonstrated that the induction of PE 1.95 with this dose of polystyrene microparticles produced a peak reduction in mean arterial blood pressure (MAP) of approximately 25% from basal measurements followed by partial recovery of arterial blood pressure to ∼10% below basal level. This dose also causes the *in vivo* RV systolic blood pressure to increase from 30 mmHg at baseline to 55 mmHg measured 30 min after embolization, suggesting ∼75% pulmonary vascular occlusion[11], [25].

PE increases pulmonary vascular resistance and RV afterload by obstructing the pulmonary arteries. With elevated RV pressures, the IVS shifts towards the LV. This shift, with concurrent pericardial constraint, reduces LV preload through interventricular interdependence and leads to impairment of the systemic and pulmonary circulation[26]. The increased gene expression of BNP likely reflects the acute hemodynamically significant right heart strain with contractile dysfunction and decreased cardiac output (CO) due to the PE. The decreased gene expression of BNP in the LV is probably due to a likely decrease in left sided pressure of the heart.

It is well known that myocardial wall stress is a stimulus for synthesis and secretion of BNP in acute or chronic LV dysfunction[27]. However there is accumulating evidence that plasma concentration of BNP is associated with PE especially when resulting in RVD. In addition increased plasma level of BNP is a significant predictor of all cause and short term mortality, also in normotensive patients and may be used for risk stratification in PE patients[28]. With this study we have shown that there exists an association between the severity of pulmonary occlusion and thereby RVD in PE and the level of BNP gene expression.

Most studies investigating the neurohormonal activation in PE have focused on BNP, however ANP is released in response to chronic increase in pulmonary artery pressure in patients with pulmonary hypertension[29] and few studies have concentrated on the role of ANP in PE[30]. To our knowledge this is the first time it is shown that ANP is up-regulated in the RV in PE, however the secretion of ANP mainly comes from the atria which have lower mass. This could explain why the plasma concentration of ANP lacks the sensitivity of BNP plasma level in relation to PE.

In the present study we found a close correlation between PE degree and gene-expression of ANP, and BNP in the cardiac chambers with a selective increase in the right chambers of the heart.

The present data supports the idea of natriuretic peptides as sensitive biomarkers of RVD in PE.
